# Breakdown of chiral recognition of amino acids in reduced dimensions

**DOI:** 10.1038/s41598-020-73300-z

**Published:** 2020-09-30

**Authors:** Yongchan Jeong, Hyo Won Kim, JiYeon Ku, Jungpil Seo

**Affiliations:** 1 Department of Emerging Materials Science, DGIST, Daegu, 42988 Korea; 2 Samsung Advanced Institute of Technology, Suwon, 16678 Korea

**Keywords:** Scanning probe microscopy, Surface assembly

## Abstract

The homochirality of amino acids in living organisms is one of the great mysteries in the phenomena of life. To understand the chiral recognition of amino acids, we have used scanning tunnelling microscopy to investigate the self-assembly of molecules of the amino acid tryptophan (Trp) on Au(111). Earlier experiments showed only homochiral configurations in the self-assembly of amino acids, despite using a mixture of the two opposite enantiomers. In our study, we demonstrate that heterochiral configurations can be favored energetically when l- and d-Trp molecules are mixed to form self-assembly on the Au surface. Using density functional theory calculations, we show that the indole side chain strongly interacts with the Au surface, which reduces the system effectively to two-dimension, with chiral recognition disabled. Our study provides important insight into the recognition of the chirality of amino acid molecules in life.

## Introduction

Amino acids containing *α*-amino (–NH_2_) and *α*-carboxyl (–COOH) groups are essential constituents for the synthesis of peptides in a living organism. Except for glycine, the amino acids exist as chiral molecules in two forms, the l- and d-enantiomers. Although the two enantiomers exhibit identical physical and chemical properties, living organisms use only amino acids of l-chirality. This homochirality of the amino acids is one of the great mysteries that need to be explored in life phenomena^1^. The amino acids are spontaneously self-assembled in space as a result of various non-covalent interactions between the molecules. For understanding the inherent chirality of the self-assembled amino acids, scanning tunnelling microscopy (STM) has been widely used^2–19^. Kühnle et al. studied the self-assembly of cysteine molecules on a Au(110) surface^5^. The result showed that homochiral cysteine dimers were stabilized by the formation of three bonds between the same enantiomers. Lingenfelder et al. investigated the interaction between l-phenylalanine (Phe)–l-Phe and d-Phe–d-Phe pairs and found that stereoselectivity plays a crucial role in the formation of the homochiral chains^12^. These pioneering works highlighted the role of chiral recognition in the self-assembly of amino acids.

**Figure 1:**
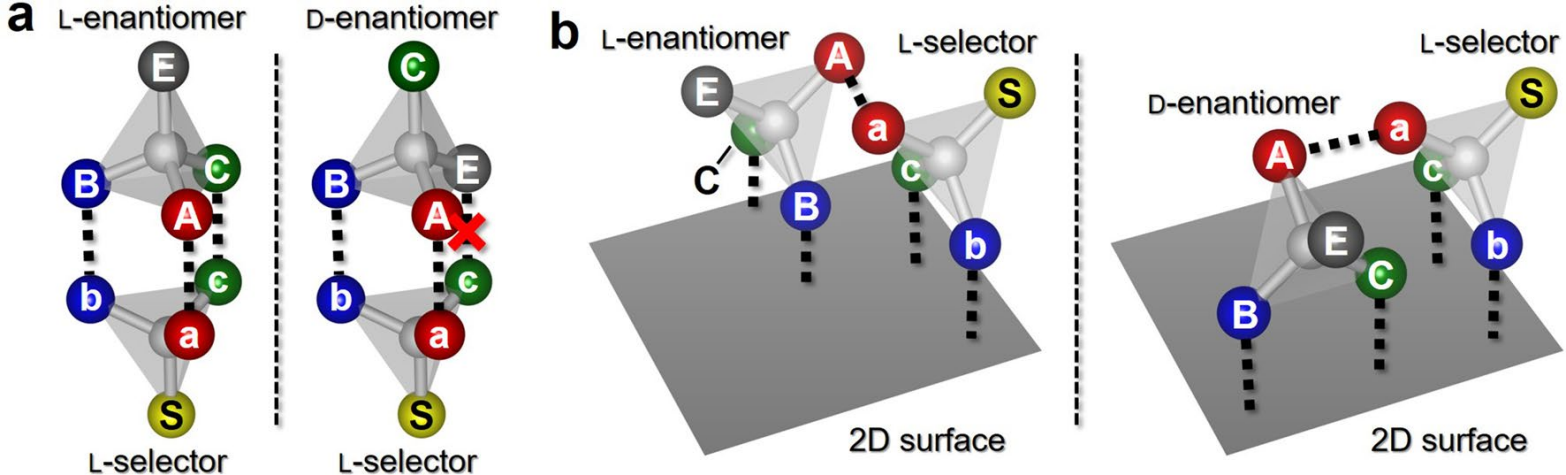
(**a**) A schematic view of the molecule–molecule interactions based on the three-point interaction model, depending on their molecular chirality. ‘A’, ‘B’, and ‘C’ sites interact with the ‘a’, ‘b’, and ‘c’ sites, respectively. (**b**) A model of the molecule–molecule interactions on a 2D surface. Only one intermolecular bond is active, between ‘A’ and ‘a’ site, owing to the interactions between the molecules and the surface.

The chiral recognition of molecules is generally discussed in terms of the three-point interaction (TPI) model, derived from the lock-and-key model^4,5,12,20–29^. Figure 1a shows the illustration of the TPI model. The l-selector uniquely mates with the l-enantiomer when bonds between the molecules are formed at three points (Fig. 1a, left panel). In contrast, the l-selector does not mate firmly with the d-enantiomer because the number of bonds is less than three (right panel). Therefore, the l-selector is energetically favored to connect to the l-enantiomer. Although the TPI model oversimplifies the nature of this process, it provides insight into the origin of the chiral recognition of chiral molecules. Dynamic conformational adjustments between the molecules help to ensure the chiral self-assembly even when the three-point bonds are not precisely defined^13,30–38^.

Since chiral recognition is based on stereochemical interaction between the chiral molecules, it is most prominent in the self-assembly in 3-dimensional (3D) space. When the number of dimensions of the system is reduced, the stereochemical interaction is limited. Figure 1b illustrates chiral molecules in 2D space. The molecules are confined to a surface as a result of interactions with the surface. We assume here that only one bond is active in the intermolecular bonding. Unlike the 3D-case in Fig. 1a, there is then no energy difference between an l-selector–l-enantiomer pair (Fig. 1b, left panel) and an l-selector–d-enantiomer pair (right panel), implying that chiral recognition is lost. As a rule, the chirality of self-assembly is significantly affected by dimensions of the system. However, thus far, no experiments have been conducted to determine if chiral recognition can be disabled in lower dimensions. In this study, we have investigated the self-assembly of tryptophan (Trp) molecules with attention to the molecular chirality, using STM. We first deposited a single type (l- or d-) of Trp molecules on a Au(111) substrate, which naturally resulted in homochiral self-assembly. In contrast, when both l- and d-Trp molecules were deposited on the substrate simultaneously, we found that heterochiral structures were predominantly formed, providing evidence of the chiral recognition being broken. By carefully modeling the self-assembled structures using density functional theory (DFT), we demonstrate that the interactions between the Trp molecules and the surface effectively reduces the system to 2D, inducing a breakdown of chiral recognition in the self-assembly of the mixture of l- and d-Trp molecules.

## Results

**Figure 2:**
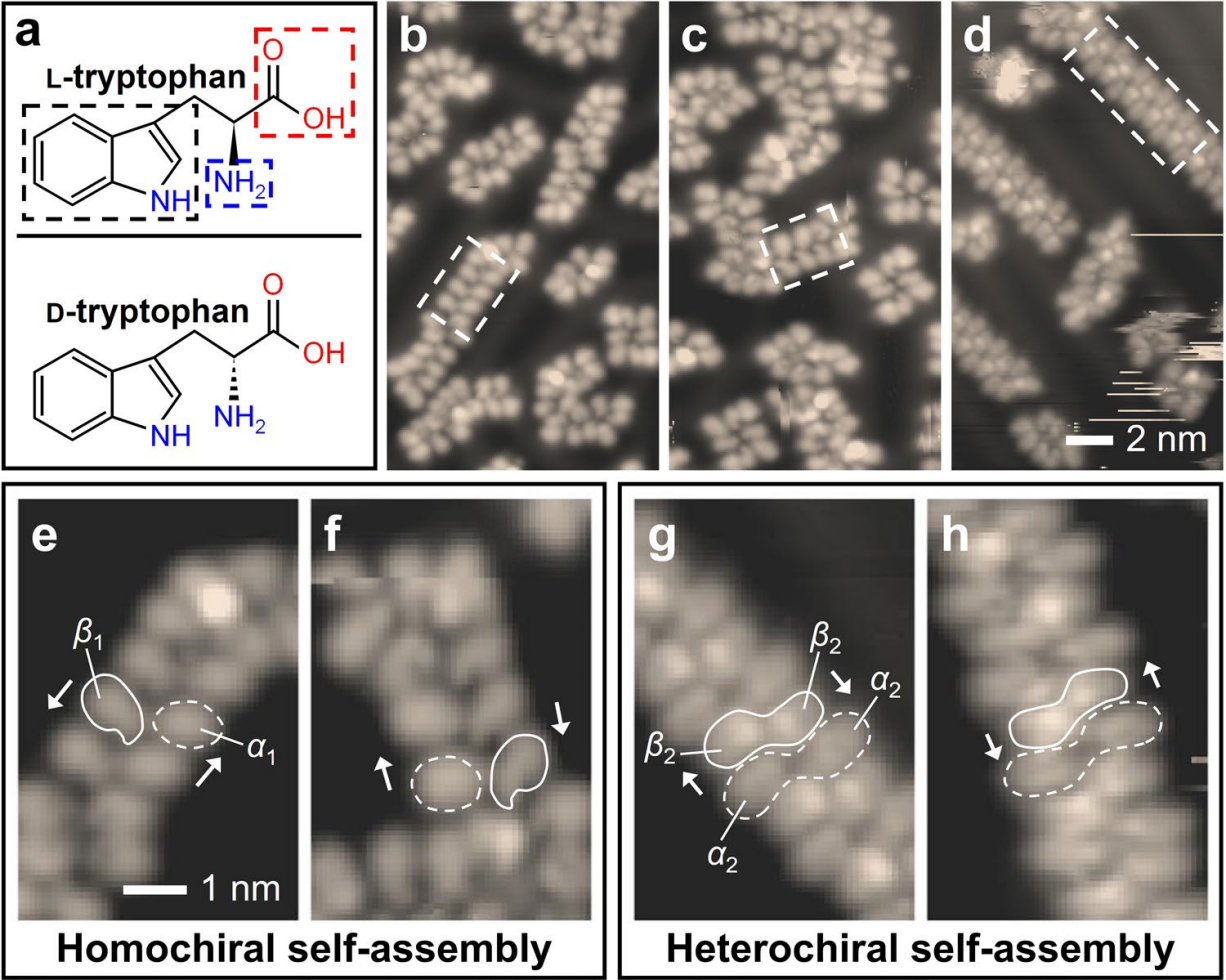
(**a**) Chemical structures of l- and d-Trp molecules. The blue, red, and black-dashed boxes indicate *α*-amino group, *α*-carboxyl group, and indole side chain, respectively. Topographic images of homochiral self-assemblies of (**b**) l- and (**c**) d-Trp molecules with a 1D chain-like structure (white-dashed box) on Au(111) surface at 77 K, with a bias voltage (V_bias_) of − 0.5 V and a tunnelling current (I_t_) of 50 pA. (**d**) The heterochiral self-assembly obtained by the deposition of a racemic mixture of l- and d-Trp molecules (V_bias_ = − 1 V, I_t_ = 50 pA). Zoom-in images of the self-assembled chain structures constructed by Trp molecules: (**e**) the l-Trp molecule-constructed and (f) the d-Trp molecule-constructed chain structures. There are two different shapes of adsorbed molecules; ellipse shape (dashed outline, *α*_1_) and apostrophe shape (solid outline, *β*_1_). (**g**, **h**) Heterochiral chain structures in the self-assembly of a racemic mixture of l- and d-Trp molecules. The dashed-outline (*α*_2_) and the solid-outline (*β*_2_) represent pairs of Trp molecules that appear to be loosely and tightly connected in the image, respectively.

A molecule of Trp, which is one of the essential amino acids used in proteins, contains an *α*-amino group, an *α*-carboxyl group, and an indole side chain, as shown in Fig. 2a. According to the bond direction of the *α*-amino group, Trp molecules are classified into l- or d-enantiomers (Fig. 2a). In the experiment, we deposited l-Trp molecules on the Au(111) surface and observed 1D-like chain structures constructed in the self-assembly (Fig. 2b). Because only l-Trp molecules are used in the self- assembly, the chain structures are naturally homochiral. The deposition of d-Trp molecules on the Au(111) surface led to the formation of similar chain structures but with the opposite chirality (Fig. 2c). This chiral-selective self-assembly motivated us to deposit the l- and d-Trp molecules in equal proportions on a clean Au(111) surface. Remarkably, the homochiral chain structures were not observed in the self-assembly, but heterochiral chain structures were observed, as shown in Fig. 2d, a fact that is confirmed in our DFT calculation.

To understand the chirality of self-assembled structures, we studied the conformation of the Trp molecules in the chain structures. Figure 2e depicts an enlarged image of the chain structure of the l-Trp molecules. Two differently-shaped l-Trp molecules are identified in the chain structure; the molecules that resembled the shape of an ellipse (dashed outline) and an apostrophe (solid outline) are denoted as *α*_1_ and *β*_1_ in Fig. 2e, respectively. The *α*_1_ and the *β*_1_ molecules form a pair, and such pairs are repeatedly connected along the chain. The structure formed by d-Trp molecules is similar to that formed by l-Trp molecules, as shown in Fig. 2f. However, their chirality is the opposite, reflecting the chirality of the individual l- and d-Trp molecules. Figure 2g,h show the chain structures formed by the racemic mixture of l- and d-Trp molecules. Two different ellipse-shaped Trp molecules are found; the *α*_2_ shape is similar to the *α*_1_ shape, but the *β*_2_ shape is a new type (Fig. 2g). The two *β*_2_ molecules are closely bound, as indicated by the solid-outline. In contrast, the two *α*_2_ molecules are loosely combined into a pair (the dashed-outline). In the self-assembly of the racemic mixture of l- and d-Trp molecules, we observed two types of chain structures with similar shapes but opposite chirality (Fig. 2g,h), indicating that they are heterochiral. The heterochirality of the racemic mixture is further supported by the cluster structures in Supplementary Information.

**Figure 3:**
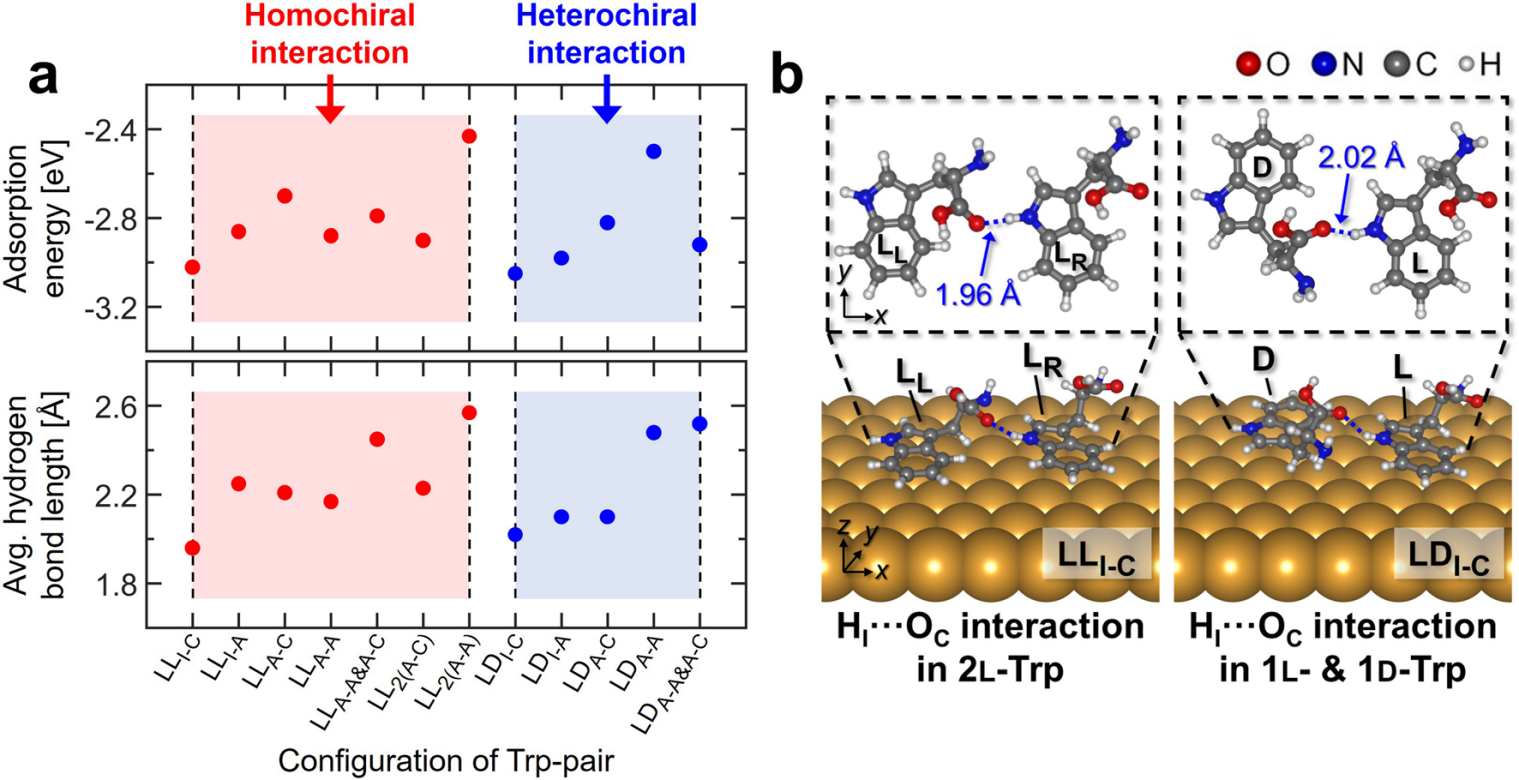
(**a**) The adsorption energy and the average hydrogen bond length in a pair of two Trp molecules on Au(111) surface. ab_*M*-*N*_ indicates the interaction between the *M* group of an a-Trp molecule and the *N* group (*M*, *N* = A: *α*-amino, C: *α*-carboxyl, I: indole) of a b-Trp molecule (a, b = l, d chirality). ab_2(*M*–*N*)_ means double hydrogen bonding. The structural detail of each Trp molecular pair is given in Supplementary Information. (**b**) Perspective and top views of the optimized 2l-Trp and the optimized 1l- & 1d-Trp configurations (the ll_I-C_ and ld_I-C_ pairs, respectively).

To understand the chain structures at the atomic scale, we investigated the Trp molecular interactions using DFT calculation. Based on the Bader charges of atoms in an l-Trp molecule (Table S1), we designed the various configurations of two Trp molecular interactions (Fig. S1). The adsorption energy (E_ads_) and the hydrogen bond (H_*M*_···X_*N*_) length for a given configuration are summarized in Fig. 3a, where X_*N*_ indicates the X atom (X = H, N, O) in the *N* group (*M*, *N* = A: *α*-amino, C: *α*-carboxyl, I: indole). ab_*M*-*N*_ is the H_*M*_···X_*N*_ interaction between the a- and b-Trp molecules (a, b = l, d chirality). E_ads_ was calculated from the equation: E_ads_ = E_*n* × Trp on Au(111)_ – (*n* × E_Trp_ + E_Au(111)_), where E_*n* × Trp on Au(111)_, E_Trp_, and E_Au(111)_ are the energies of the *n* × Trp molecules adsorbed on Au(111), the isolated Trp molecule, and the pure Au(111), respectively. In the pair of two l-Trp molecules, the lowest E_ads_ was found to be − 3.02 eV (ll_I-C_, left panel in Fig. 3b) when the O_C_ atom in an l-Trp molecule was close to the H_I_ atom in the other l-Trp molecule (H_I_···O_C_ interaction). In the pair of l- and d-Trp molecules, a similar H_I_···O_C_ interaction leads to the lowest E_ads_ of − 3.05 eV (ld_I-C_, the right panel in Fig. 3b). Although the H_I_···O_C_ distance of the ll_I-C_ pair is shorter than that of the ld_I-C_ pair, the ll_I-C_ pair exhibited a slightly higher E_ads_. To understand this, we investigated the strain energies and the H_I_^*b*^-N_I_-C_I_^*b*^ angles in Trp molecules of the ll_I-C_ and ld_I-C_ pairs (Table S2), where H_I_^*b*^ and C_I_^*b*^ are the H_I_ and C_I_ atoms bonded to the N_I_ atom in a Trp molecule. The right-hand l-Trp molecule (l_R_) in the ll_I-C_ pair produced extra strain energy, owing to the change in the H_I_^*b*^-N_I_-C_I_^*b*^ angle, which is responsible for the higher formation energy of the ll_I-C_ pair.

**Figure 4:**
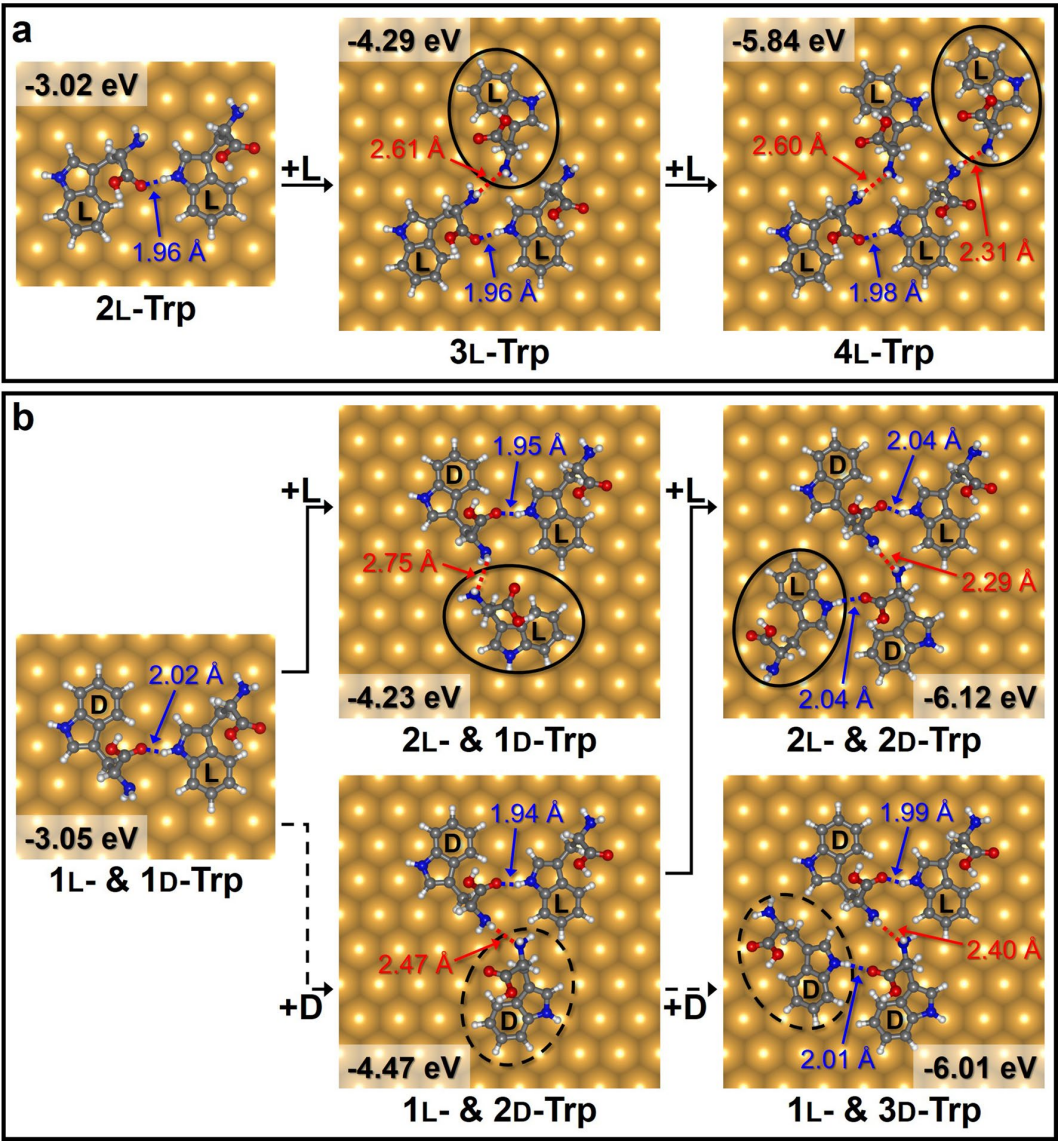
Construction process of (**a**) homochiral and (**b**) heterochiral structures consisting of four Trp molecules, with the adsorption energies shown. The black solid and dashed ovals indicate l- and d-Trp molecules, respectively. The blue and red dotted lines are the H_I_···O_C_ and H_A_···N_A_ bonds, respectively.

We constructed a structure of more than two Trp molecules by adding Trp molecules to the configurations found in Fig. 3b. Figure 4a shows the construction of a chain of l-Trp molecules. We attached an l-Trp molecule next to the H_I_···O_C_ bond of the ll_I-C_ pair. When the N_A_ atom in the l-Trp molecule (black solid oval) is close to the H_A_ atom near the H_I_···O_C_ bond of the ll_I-C_ pair, the 3l-Trp configuration is optimized with an E_ads_ of − 4.29 eV. In a subsequent calculation, we added another l-Trp molecule to the 3l-Trp configuration. The H_A_···N_A_ interaction between the added l-Trp molecule and the 3l-Trp configuration resulted in the optimized 4l-Trp configuration with an E_ads_ of − 5.84 eV.

The racemic mixed chain structures were also investigated, as shown in Fig. 4b. In separate calculations, l- or d-Trp molecules were added to the stable 1l- & 1d-Trp configuration (the ld_I-C_ pair shown in Fig. 3b). When the N_A_ atom of the added l-Trp molecule was close to the H_A_ atom of the d-Trp molecule in the ld_I-C_ pair, the 2l- & 1d-Trp configuration was optimized with an E_ads_ of − 4.23 eV. The addition of a d-Trp molecule to the ld_I-C_ pair gave the optimized 1l- & 2d-Trp configuration resulting from the H_A_···N_A_ interaction between the added d-Trp molecule (black dashed oval) and the d-Trp molecule in the ld_I-C_ pair, with a H_A_···N_A_ distance of 2.47 Å. The optimized configuration required an E_ads_ of − 4.47 eV, which is the lowest E_ads_ among the possible structures consisting of three Trp molecules, because it has the shortest H_A_···N_A_ distance.

**Figure 5:**
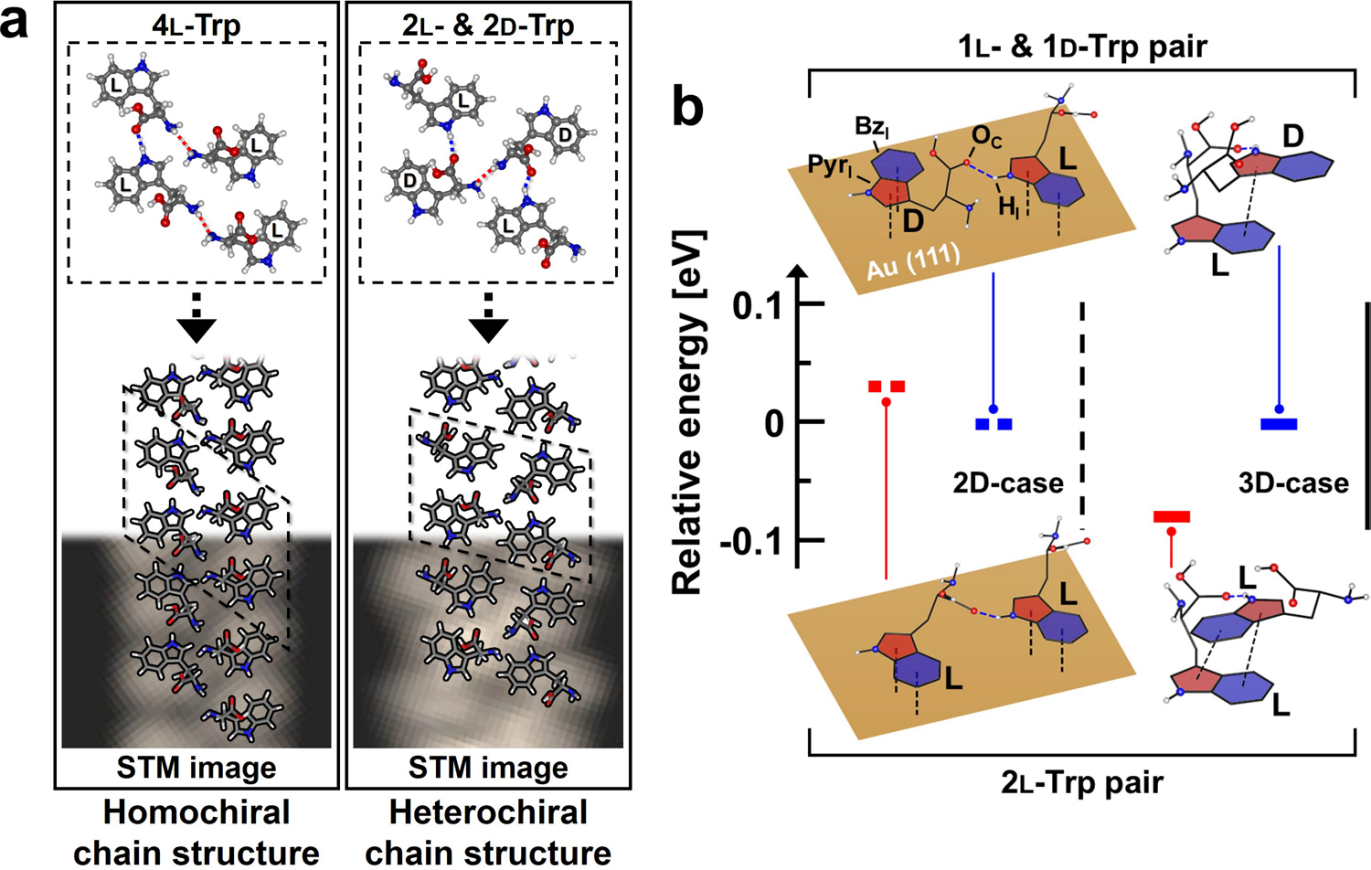
(**a**) Modelling of continuous 1D-chain structures using the optimized configurations consisting of four Trp molecules (black-dashed parallelogram as a periodic unit cell), with the STM-measured chain structures. (**b**) The energy difference between the homochiral (2l-Trp pair) and the heterochiral (1l- & 1d-Trp pair) structures in the 2D-case (on surface) and 3D-case (in vacuum). The energies of 2l-Trp pairs are referenced to those of 1l- & 1d-Trp pairs.

By adding l- or d-Trp molecules to the optimized 1l- & 2d-Trp configuration, we constructed heterochiral structures consisting of four Trp molecules. When the H_I_ atom of the added d-Trp molecule approached the O_C_ atom of the d-Trp molecule that did not participate in the H_I_···O_C_ interaction, the 1l- & 3d-Trp configuration was optimized with an E_ads_ of − 6.01 eV. However, when an l-Trp molecule was added to the optimized 1l- & 2d-Trp configuration, the additional H_I_···O_C_ interaction gave rise to the optimized 2l- & 2d-Trp configuration with an E_ads_ of − 6.12 eV. This energy is lower than the E_ads_ of the optimized 1l- & 3d-Trp configuration owing to the shorter total hydrogen bond length. The optimized 2l- & 2d-Trp configuration had two H_I_···O_C_ and one H_A_···N_A_ interactions, whereas the optimized 4l-Trp configuration consisted of one H_I_···O_C_ and two H_A_···N_A_ interactions. As a result, the optimized 2l- & 2d-Trp configuration showed a lower E_ads_ than the optimized 4l-Trp configuration because the optimized 2l- & 2d-Trp configuration included more H_I_···O_C_ interactions, which are more stable than H_A_···N_A_ interactions. The result that the heterochiral configuration was energetically favorable than the homochiral configurations was in agreement with our STM results (Fig. 2d). By using the optimized configuration of 4l-Trp and the 2l- & 2d-Trp molecules as a periodic unit cell, we designed continuous chain structures, as shown in Fig. 5a. Geometrically, the chain structures constructed using the configurations optimized by DFT calculation were in good agreement with the experimentally obtained STM images.

## Discussion

Previous studies have shown that homochiral structures are assembled even if a mixture of l- and d-amino acids is deposited on substrates, which indicates that the chiral recognition of amino acids is in play^5,12,13^. However, this is not necessary in nature, as is demonstrated in our experiments. To understand the breakdown of chiral recognition in our system, we consider the role of the Au(111) surface in the self-assembly. The Trp molecules are adsorbed on this surface by interactions between the indole groups of the molecules and the Au(111) surface. The indole group comprises pyrrole-like (Pyr_I_) and benzene-like (Bz_I_) rings; thus, the energy optimization of the 2l-Trp and 1l- & 1d-Trp pairs is mainly governed by the three interactions: H_I_···O_C_, Pyr_I_···Au, and Bz_I_···Au. Among them, the Pyr_I_ and Bz_I_ rings of the Trp molecule interact strongly with the Au(111) surface. The intermolecular interaction is then guided only by the H_I_···O_C_ bonding (refer to the Trp molecules in the 2D-case in Fig. 5b), a proof of the concept depicted in Fig. 1b. Hence, it is concluded that the strong interaction between the Trp molecules and the Au(111) surface reduces the system to 2D, with the chiral recognition disabled. To understand how the lock-and-key model in Fig. 1a works for Trp molecules in 3D space, we calculated the interaction between two Trp molecules in the absence of the Au(111) surface. The 2l-Trp molecules are optimized in energy by two Pyr_I_···Bz_I_ (*π*–*π* stacking) interactions and one H_I_···O_C_ interaction (refer to the Trp molecules in the 3D-case in Fig. 5b). In contrast, the 1l- and 1d-Trp molecules are restricted to the configuration with one Pyr_I_···Bz_I_ interaction and one H_I_···O_C_ interaction, owing to their steric hindrance. As a result, the energy of the 2l-Trp molecules is lower than that of the 1l- and 1d-Trp molecules, a fact that was postulated as the origin of the chiral recognition^20–29,39–42^.

In this study, we demonstrated the heterochiral self-assembly of Trp molecules on a Au(111) surface by STM measurements. The deposition of two opposite Trp enantiomers on the Au(111) surface led to a heterochiral chain structure, which differs from the homochiral chain structure obtained from single Trp enantiomers. To understand the construction mechanism of the heterochiral structure, we performed a DFT calculation. Trp molecules were adsorbed on a Au(111) surface, with interaction occurring between the indole group of the Trp molecule and the Au(111) surface. This adsorption condition causes the dimension of system to be effectively reduced to 2D in the self-assembly. As a result, only a limited H_I_···O_C_ bonding contributes to the molecule–molecule interaction and the chiral recognition of Trp molecules is broken. Our work provides insight into the self-assembly of chiral molecules in low dimensions and deepens our understanding of how and when the chiral recognition can be disabled in our body.

## Methods

### STM measurements

All experiments were carried out using a home-built variable-temperature STM with a base pressure of 8 × 10^−11^ Torr. We used a mechanically sharpened Pt-Ir tip for obtaining STM images. The Au(111) substrate was cleaned by repeated cycles of Ar ion sputtering at an Ar pressure of 1.5 × 10^−5^ Torr (10 min) alternating with annealing at 600 °C (15 min). The Trp molecules (Sigma-Aldrich l-Trp: T0254 and d-Trp: T9753) were deposited on the Au(111) surface using two Knudsen cells; one for l-Trp and the other for d-Trp. Both Knudsen cells were used for the deposition of the racemic mixture of l-Trp and d-Trp. The substrate temperature was maintained at 100 °C during the deposition. The sample was then cooled to 77 K for the STM measurements.

### DFT calculations

The initial atomic coordinates of a single Trp molecule for the DFT calculation were obtained from ‘https://pubchem.ncbi.nlm.nih.gov/compound/Tryptophan’. This structure is most stable in its isolated form (Supplementary Information). The DFT calculation was performed using the Vienna *Ab-initio* Simulation Package (VASP) code^43,44^, employing the project augmented wave (PAW) method of Blöchl^45^ implemented in VASP by Kresse and Joubert^46^. The exchange correlation energy was determined by means of the generalized-gradient approximation (GGA) of Perdew, Burke, and Ernzerhof (PBE) ^47^. All energies were acquired by considering a non-local van der Waals density functional^48^ and optPBE^49,50^. A cutoff energy of 500 eV and 2 × 2 × 1 k-points mesh with zero shift for a slab structure containing a vacuum layer with a thickness of 25 Å were used to generate a plane-wave basis set by the Monkhorst–Pack grid method. Electronic and geometry optimizations were converged when the total energy difference between successive calculation steps was less than 10^−4^ and 10^−3^ eV, respectively. All atoms were allowed to relax until the force on each ion was less than 0.02 eV/Å. The Bader analysis method was performed to calculate the electronic charge density of atoms in a Trp molecule^51,52^. We constructed four-layer Au(111) slabs in 9 × 9 and 10 × 10 super cells using the optimized Au(111) unit cell to investigate the interactions between more than 2 Trp molecules (9 × 9 Au(111) slab: two or three Trp molecules; 10 × 10 Au(111) slab: four Trp molecules). Only the two top layers in the Au(111) slabs were allowed to relax.

### Data availability

The data that support the findings of this study are available from the corresponding author upon reasonable request.

## Supplementary information

Supplementary Information 1.
