# Supplementary material for: Breakdown of chiral recognition of amino acids in reduced dimensions
Source: Sci Rep. 2020 Sep 30;10:16166. doi: 10.1038/s41598-020-73300-z (PMC7527561; doi:10.1038/s41598-020-73300-z)
Supplement: Supplementary file 1 — Supplementary Information 1. [file 41598_2020_73300_MOESM1_ESM.pdf]

Supplementary Information

Breakdown of Chiral Recognition of Amino Acids in Reduced Dimensions

Yongchan Jeong[1,*], Hyo Won Kim[2], Jiyeon Ku[2], and Jungpil Seo[1,*]

[1]*Department of Emerging Materials Science, DGIST, Daegu 42988, Korea*

[2]*Samsung Advanced Institute of Technology, Suwon 16676, Korea*

*Address correspondence to ycjeong@dgist.ac.kr or jseo@dgist.ac.kr

Table S1. Bader charges of atoms in an L-tryptophan (Trp) molecule. X_N indicates the X atom (X = H, N, O) in the *N* group (*N* = A: *α*-amino, C: *α*-carboxyl, I: indole). X_I^b indicates the X atom bonded to the N atom in the indole side chain of Trp molecule (X = H, C).

Group	Atom	Bader charge
α-amino (Blue-dashed oval)	N_A	–1.01
	H_A	+0.36, +0.38
α-carboxyl (Red-dashed oval)	C_C	+1.46
	O_C	–1.12, –1.10
	H_C	+0.61
Indole (Black-dashed oval)	C_I	–0.09 ~ +0.06
	C_I^b	+0.34, +0.35
	N_I	–1.19
	H_I	+0.02 ~ +0.04
	H_I^b	+0.43

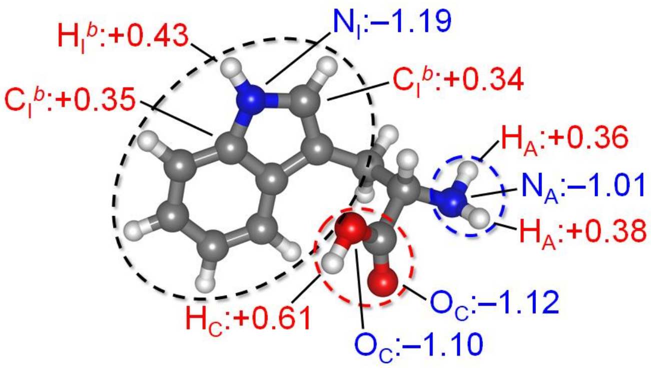

Table S2. The strain energies and the H_I^b-N_I-C_I^b angles of Trp molecules in the $LL_{I\text{-}C}$ and $LD_{I\text{-}C}$ pairs shown in Figure 3b. L_L and L_R are the left and right L-Trp molecule in the $LL_{I\text{-}C}$ pair, respectively.

Pair	Chirality	Strain energy	H_I^b-N_I-C_I^b angle
$LL_{I\text{-}C}$	L_L	0.02 eV	125 ^{o}
	L_R	0.04 eV	123, 126 ^{o}
$LD_{I\text{-}C}$	D	0.02 eV	125 ^{o}
	L	0.02 eV	124, 126 ^{o}
Intact		0	125, 126 ^{o}

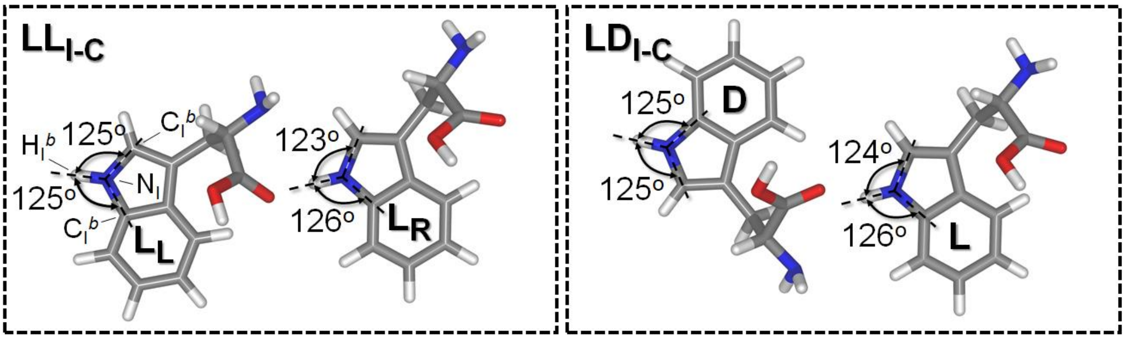

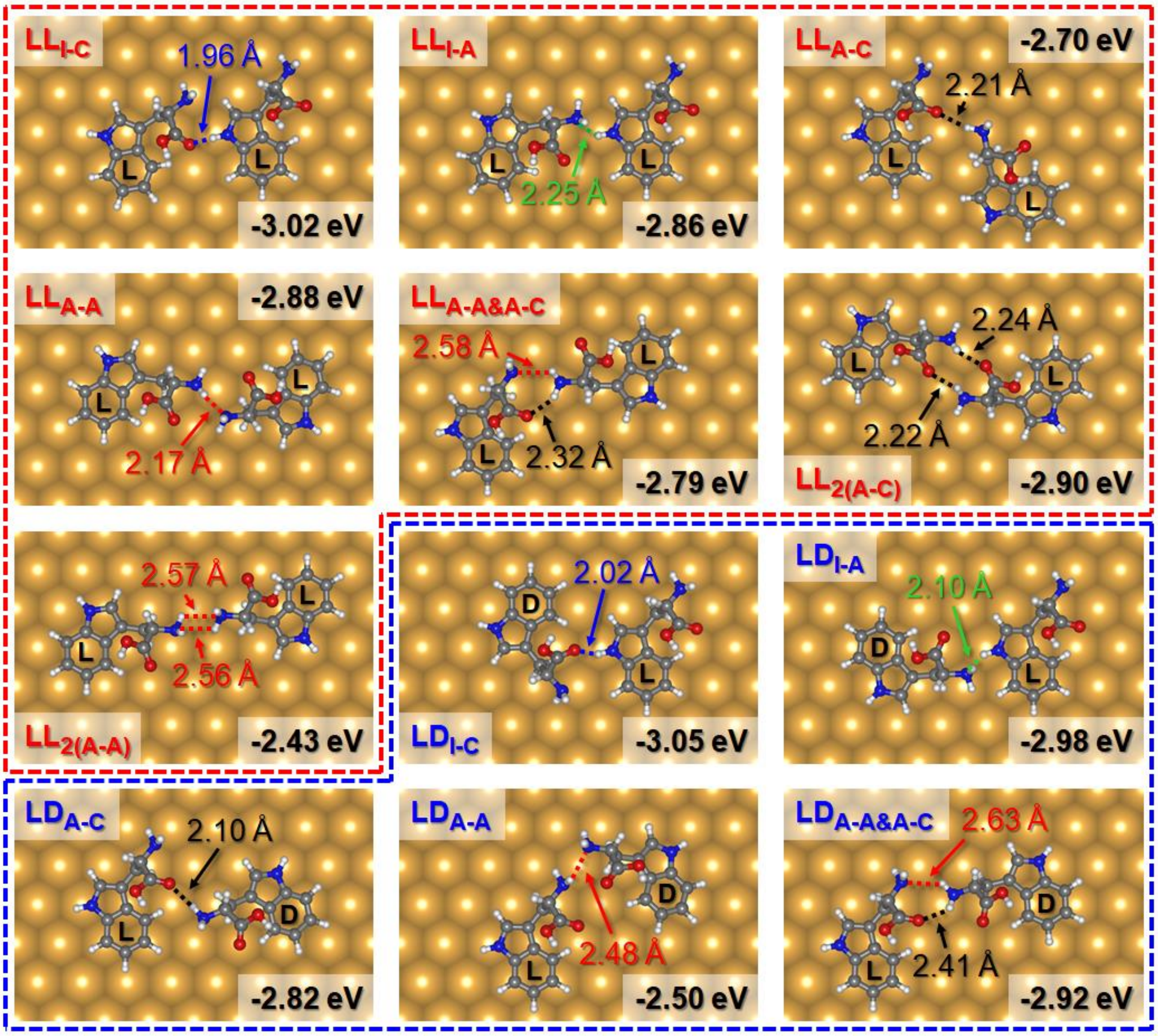

Figure S1. Configurations of Trp molecular pair on Au(111) surface. The red- and the blue-dashed regions indicate the pairs of L- and L-Trp molecules and the pairs of L- and D-Trp molecules, respectively. $AB_{M\text{-}N}$ indicates the interaction between the *M* group of an A-Trp molecule and the *N* group (*M*, *N* = A: *α*-amino, C: *α*-carboxyl, I: indole) of a B-Trp molecule (A, B = L, D-chirality). $AB_{2(M\text{-}N)}$ means double hydrogen bonding.

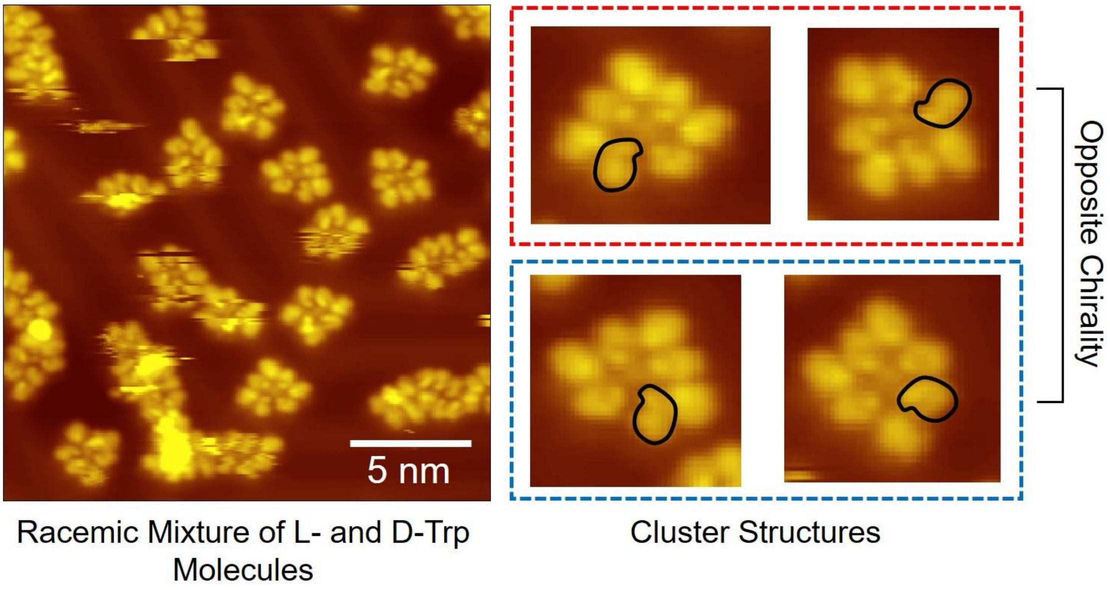

Figure S2. The chirality of the cluster structures. In addition to the chain structures shown in main text, we observe cluster structures in the self-assembly of Trp molecules. In the racemic mixture of L- and D-Trp molecules, the cluster structures of opposite chirality are observed at the same time in the image, which reveals that they are heterochiral structures.

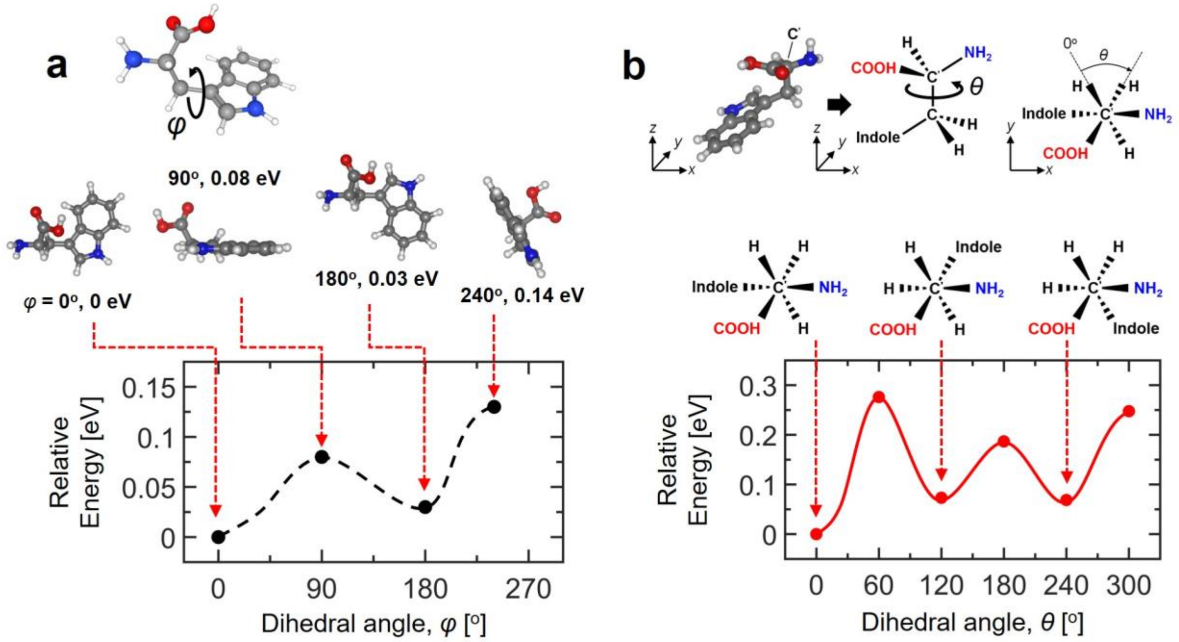

Figure S3. Relative energy of a Trp molecule with the functional groups rotated. (a) Energy as a function of the rotation angle of the indole group. (b) A perspective view and chemical structures of the Trp molecule. C' indicates the C atom bonded to α-amino (–NH2) and α-carboxyl (–COOH) groups. (b) Energy as a function of angle *θ*.
